# Cardiopulmonary transit time measured on dynamic rubidium cardiac PET/CT is a predictor for pulmonary hypertension

**DOI:** 10.1186/s13550-026-01387-y

**Published:** 2026-06-22

**Authors:** Lena C. Seige, Jakob Heimer, Karsten Murray, Eva Seiler, Niklas Stauffer, Cristina Popescu, Christian la Fougère, Eric Moulton, Nidaa Mikail, Sabin G. Pop, Irene A. Burger, Alexander W. Sauter

**Affiliations:** 1 Department of Nuclear Medicine, Cantonal Hospital Baden, Partner hospital for research and teaching of the Medical Faculty of the University of Zurich, Im Ergel 1, Baden, 5404 Switzerland; 2 ETH Zurich, Digital Trial Innovation Platform, Partnerhaus KSB, Baden, 5404 Switzerland; 3 Department of Cardiology, Cantonal Hospital Baden, Baden, 5404 Switzerland; 4 Department of Internal Medicine, Cantonal Hospital Baden, Baden, 5404 Switzerland; 5 Cardiovascular Research Institute Basel, University Hospital Basel, Basel, 4056 Switzerland; 6 Department of Nuclear Medicine and Clinical Molecular Imaging, University Hospital Tuebingen, 72074 Tuebingen, Germany; 7 Jubilant Radiopharma, Kirkland, Québec Canada; 8 Department of Nuclear Medicine, University Hospital Zurich, University of Zurich, Zurich, 8006 Switzerland; 9 Department of Health Sciences and Technology, ETH Zurich, Zurich, 8092 Switzerland; 10 Department of Radiology, University Hospital Tuebingen, 72076 Tuebingen, Germany

**Keywords:** Positron emission tomography computed tomography, Rubidium-82, Myocardial perfusion imaging, Echocardiography, Pulmonary transit time, Pulmonary hypertension

## Abstract

**Background:**

Cardiopulmonary transit time (CPTT), the time for blood to circulate from the right to the left ventricle, can be assessed on dynamic Rubidium-82 ([^82^Rb]) cardiac PET/CT. Given its association with cardiac and pulmonary function, CPTT holds potential as a screening marker for pulmonary hypertension. This study investigated the relationship between CPTT and echocardiographic markers of cardiac function, as well as its association with pulmonary hypertension. In this retrospective single-center study, 111 patients (72 male, 39 female) referred for [^82^Rb]RbCl-PET/CT and echocardiography within 31 days were included. CPTT, normalized to heart rate (NCPTT), was calculated from peak right and left ventricular [^82^Rb] activity and examined in relation to patient characteristics and echocardiographic parameters, which were further used to categorize left and right ventricular systolic dysfunction, left ventricular diastolic dysfunction, and signs of elevated pulmonary arterial pressure.

**Results:**

Prolonged NCPTT was significantly associated with lower left ventricular ejection fraction and increased body weight (*p* < 0.05). NCPTT was significantly associated with left and right ventricular systolic dysfunction (OR = 1.21, 95%CI:1.04–1.42, *p* < 0.05; OR = 1.18, 95%CI:1.04–1.34, *p* < 0.01), elevated pulmonary arterial pressure (OR = 1.21, 95%CI:1.02–1.44, *p* < 0.05), and possible pulmonary hypertension (*p* < 0.05), but not with left ventricular diastolic dysfunction.

**Conclusion:**

Our findings suggest that NCPTT may serve as a useful additional marker for assessing cardiac function, particularly ejection fraction, and could help in the evaluation of patients with suspected cardiac systolic dysfunction, as well as the detection of pulmonary hypertension.

**Supplementary Information:**

The online version contains supplementary material available at 10.1186/s13550-026-01387-y.

## Background

Pulmonary hypertension (PH) is a chronic condition affecting up to 10% of individuals over the age of 65 [1]. PH is characterized by elevated pulmonary arterial pressure (PAP), defined as a mean pulmonary arterial pressure (mPAP) > 20 mmHg at rest, as measured by right heart catheterization (RHC) [2]. Left-sided heart failure, especially heart failure with preserved ejection fraction (HFpEF), is the leading cause of PH [1]. If left untreated, PH can lead to right heart failure and death. PH‘s non-specific symptoms often result in delayed diagnosis, highlighting the need for non-invasive screening tools [2–4]. While transthoracic echocardiography (TTE) remains the primary screening tool for PH [2, 5], TTE lacks sensitivity in early or mild forms of the disease [6], underscoring the necessity for novel screening markers.

Cardiopulmonary transit time (CPTT), i.e. the time it takes for a blood bolus to travel from the right ventricle (RV) to the left ventricle (LV) through pulmonary circulation [7], is influenced by both cardiac output and pulmonary vascular resistance. Prolonged CPTT can indicate hemodynamic disturbances, such as elevated pulmonary vascular resistance, which is characteristic of pulmonary hypertension (PH). Prior imaging studies, utilizing computed tomography and cardiac magnetic resonance, have indeed demonstrated increased CPTT in PH patients, showing correlation with elevated pulmonary vascular resistance [8]. These findings suggest CPTT’s potential utility as a screening parameter for PH.

Rubidium-82 positron emission tomography/computed tomography ([^82^Rb]RbCl-PET/CT), an increasingly used method for assessing myocardial perfusion, builds on tracking the dynamic uptake of the tracer from the cardiac cavities to the myocardium. As such, [^82^Rb]RbCl-PET/CT allows assessing CPTT, and could thus help detect PH [9–12]. 

Therefore, the aim of this study was to evaluate whether cardiopulmonary transit time normalized to heart rate (NCPTT), measured by dynamic [^82^Rb]RbCl-PET/CT, using an optimized 3-second temporal resolution protocol, is associated with echocardiographic markers of right cardiac function and elevated pulmonary arterial (PA) pressure. By systematically comparing NCPTT to established echocardiographic parameters and clinical indicators of possible pulmonary hypertension in a real-world cohort, this study aims to determine the potential of NCPTT as a non-invasive biomarker for the detection of PH in routine clinical practice.

## Materials and methods

### Study design and population

**Fig. 1:**
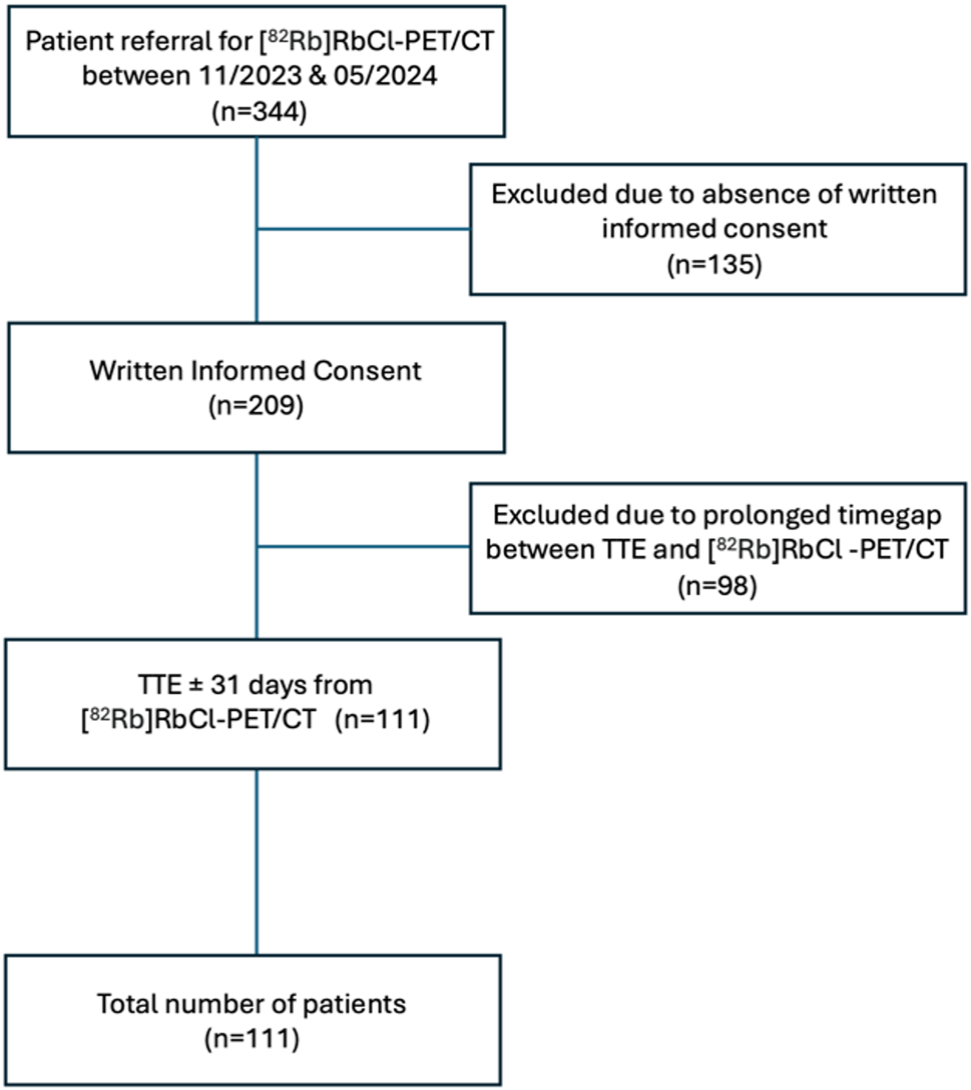
Study flow chart

This retrospective study included patients referred for [^82^Rb]RbCl-PET/CT and TTE within 31 days at the outpatient clinic of a single center (Department of Nuclear Medicine, Kantonsspital Baden, Switzerland) between November 2023 and May 2024 (Fig. 1). In comparison to the preliminary study, an adjustment was made to the imaging protocol, reducing the duration of the frameset during [^82^Rb]RbCl-PET/CT acquisition from 10 s to 3 s [12]. The aim of this modification was to improve temporal resolution and optimize image quality (see Supplementary Material). Exclusion criteria included: (i) breastfeeding or pregnancy; (ii) food intake within four hours before [^82^Rb]RbCl-PET/CT; (iii) caffeine/theine intake within twelve hours before [^82^Rb]RbCl-PET/CT. The study adhered to the ICH-GCP guidelines and the Declaration of Helsinki and was approved by the local ethics committee (2023–02188). Written informed consent was obtained from all patients.

Additionally, we applied the H_2_FPEF-Score [13](Supplementary Fig. 3) to estimate the probability of underlying HFpEF, one of the leading causes of PH [1], in our patient cohort. The score consists of six clinical variables: body mass index (BMI) > 30 kg/m^2^, age > 60 years, intake of ≥ 2 antihypertensive medications, presence of paroxysmal or persistent atrial fibrillation, an estimated sPAP > 35 mmHg and the estimated LV filling pressures with an E/e’_ratio_ > 9. While BMI, age, medication intake, and atrial fibrillation were readily available, not all patients underwent TTE. Among those who did, sPAP and E/e’_ratio_ measurements were only available in a subset of cases.

### Clinical categories

The clinical characteristics of patients were collected, including comorbidities, medication intake, and potential associations with CPTT (see Supplementary Material). As none of the patients had been diagnosed with PH by RHC, they were classified according to established guidelines, and in collaboration with an experienced in-house cardiologist as having ‘possible pulmonary hypertension’ (PPH) if any of the following signs suggestive of increased pulmonary pressure were met [2]: i) on CT scans, a truncus pulmonalis/aorta ascendens ratio (ratio tr.pulm: aorta) ≥ 1 [14]; ii) on TTE, a pressure gradient between the right ventricle and the right atrium ≥ 33 mmHg, which corresponds to an estimated mean pulmonary artery pressure (mPAP) ≥ 25 mmHg. In accordance with current ESC/ERS guidelines [2], pulmonary hypertension (PH) is defined as a mean pulmonary artery pressure (mPAP) > 20 mmHg as measured by right heart catheterization (RHC). However, since echocardiography provides only an indirect estimation of pulmonary pressures, we used an mPAP threshold of ≥ 25 mmHg as suggestive of PH.

### Imaging protocols

#### PET acquisition

[^82^Rb]RbCl-PET/CT studies were performed on a Siemens Biograph mCT Flow (Siemens Healthineers, Erlangen, Germany). Prior to the PET acquisition, a low-dose CT scan was conducted for attenuation correction and calcium scoring, following established guidelines [15]. Rubidium-Chloride ([^82^Rb]RbCl) was administered at a dose of 7.5 MBq/kg, with a maximum injected dose of no more than 1087.5 MBq (mean ± standard deviation (SD): 631 ± 142.7 MBq). In a preliminary study [12], image acquisition was performed using the vendor’s predefined 10-second framesets, a duration which determines the length of each time interval during data acquisition. However, in order to increase the time resolution and thus improve sensitivity, we shortened the framesets. Therefore, in the present study, dynamic images were reconstructed with the following modified framesets: initial 1–2 s delay, followed by 39 × 3-second frames. Reconstruction parameters included the following: reconstruction method OSEM3D + TOF 3i21s, scatter correction method, model-based, absolute scatter scaling, 220 × 220 matrix with a zoom factor of 2, yielding 1.65 × 1.65 mm pixel spacing.

[^82^Rb]RbCl was injected using the RUBY-FILL^®^ generator and [^82^Rb] elution system (Jubilant Radiopharma, Kirkland, QC, Canada) at a maximum flow rate of 30 mL/min, ensuring the shortest possible bolus infusion. Both rest and stress imaging were obtained for each patient. Stress was pharmacologically induced using adenosine, administered over 6 min at a dose of 0.14 mg/kg/min, with [^82^Rb]RbCl injected 2 min and 45 s after the start of the infusion. In cases where adenosine was contraindicated (e.g., in patients with COPD or asthma) [15], a single injection of regadenoson (Rapiscan^®^, 400mcg/5mL) was administered under the supervision of the responsible cardiologist.

### Image analysis

**Fig. 2:**
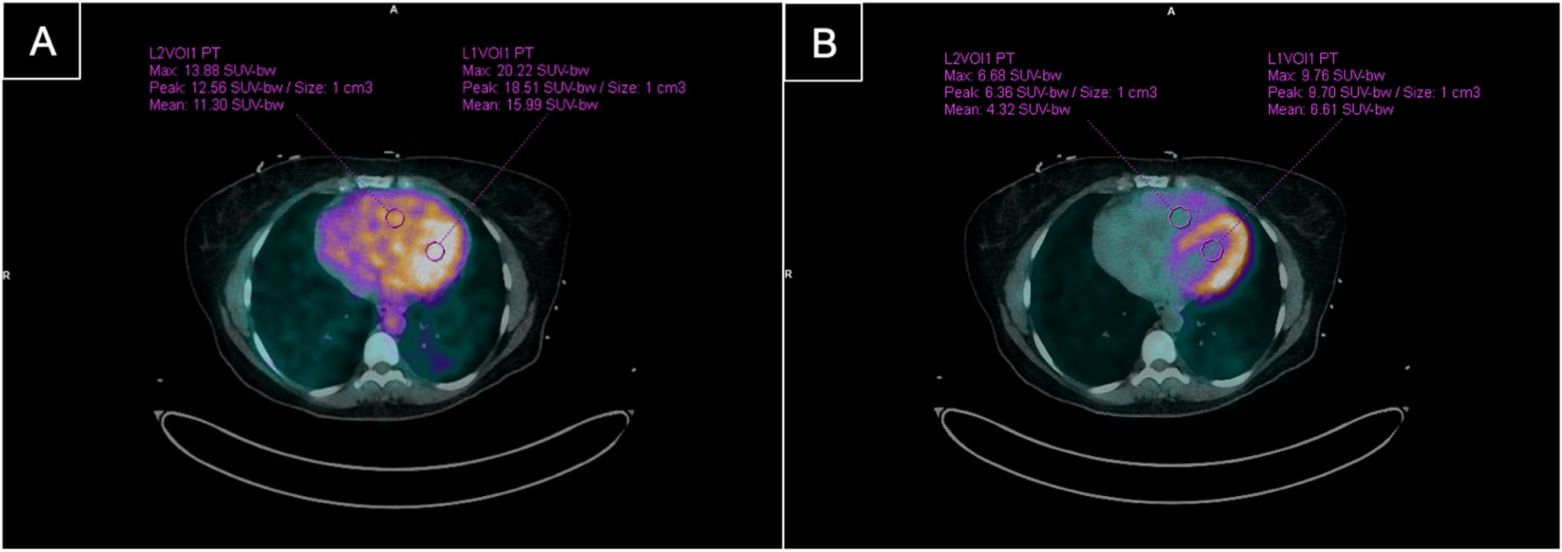
Time-activity curves were generated from PET activity in left (L1VOI1) and right (L2VOI1) ventricular cavities, identified in axial views during early (**A**) and late (**B**) phases

Image analysis was performed by a physician (L.C.S.) using standardized 1 cm³ volumes of interest (VOI) in the RV and LV on horizontal long-axis views with syngo.via (MM Oncology, version VB60A, Siemens Healthineers)(Fig. 2). A board-certified nuclear medicine physician and radiologist (A.W.S.) double-checked the analysis. Standardized uptake values (SUV), including SUV_mean_, and time-activity curves were obtained. The curves showed sharp peaks marking bolus arrival in each ventricle during first-pass perfusion. Peak isolation enabled SUV_mean_-based arrival time calculation, with the time difference used to determine the CPTT.

### Transthoracic echocardiography (TTE)

Two-dimensional and Doppler TTE were performed within 31 days of the [⁸²Rb]RbCl-PET/CT exam. Subjects were imaged in the left-lateral decubitus position using a Vivid E9 scanner (version BT11, GE Healthcare, Horten, Norway) with phased-array transducers (M5S-D and 4V-D). Parasternal long- and short-axis views and three standard apical views were acquired, with 2–3 cardiac cycles recorded during quiet respiration. LV volumes were measured from apical four- and two-chamber views using Simpson’s rule. After exclusion of echocardiographic parameters with missing data (Table 3), final parameters included LV ejection fraction (EF_Echo_), left atrial volume (LA_volume_), tricuspid annular plane systolic excursion (TAPSE), mitral inflow pattern (E and A velocities; E/A_ratio_), and estimated LV filling pressures using the E/e’_ratio_.

### Categorization of cardiac function

Additionally, TTE parameters were categorized using classifications defined by three experienced in-house cardiologists to address missing data and ensure consistent interpretation. Each case was independently reviewed by two cardiologists, with disagreements resolved through consensus discussion. The defined categories were:

1. Diastolic LV dysfunction (no, grade 1/2/3, N/A), indicating absence, presence, severity, or unavailability of data;
2. Global systolic LV dysfunction (no, yes, N/A), indicating whether dysfunction was absent/present/non-available;
3. Systolic RV dysfunction (no, yes, N/A), indicating whether right ventricular dysfunction was absent/present/non-available;
4. Signs of increased PA pressure present (no, yes).

### Statistical analysis and modeling

All statistical analyses were computed with R (4.4.1). The significance level was set at α = 0.05. Only rest NCPTT results are presented, as rest and stress NCPTT measurements highly correlate. The use of CPTT normalized to heart rate (NCPTT), rather than absolute CPTT values was chosen to reduce inter-individual variability and provide a more standardized and physiologically meaningful assessment of cardiopulmonary transit dynamics. We compared NCPTT values during rest (NCPTT_rest_) between patients with and without specific clinical characteristics, including pathologies, medical history, and cardiologic diagnoses based on TTE. NCPTT_rest_ values are reported as mean ± SD, and group comparisons were performed with a Wilcoxon rank-sum test. To test robustness, all EF measured through PET (EF_PET_) cases and CPTT in 20 random rest/stress patients (CPTT_rest+stress_) were reanalyzed by L.C.S. A multiple linear regression model was used to assess the association between resting NCPTT_rest_ as the dependent variable and the following predictors: sex, age, height, weight, EF_Echo_, LA_volume_, TAPSE, E/A_ratio_, and E/e’_ratio_. For modeling, variables included in the regression were imputed using multiple imputation by chained equations (MICE). For the types of cardiac dysfunctions, univariate logistic regression was performed with the diagnosis as the dependent variable and NCPTT_rest_ as the independent variable, and odds ratios (OR) were calculated from the model coefficients. For all linear models, model assumptions were assessed graphically with the DHARMa package. For assessing reproducibility of measurements, intraclass correlation (ICC) and Bland-Altman analysis for repeated measures were calculated for EF_Echo_ and CPTT_rest+stress_. To determine the cutoff value of NCPTT_rest_ corresponding to a sPAP of 36 mmHg, we used the linear regression equation derived from the correlation between NCPTT_rest_ and sPAP.

## Results

### Patient demographics

The patients’ characteristics, including clinical comorbidities, history and medication intake, are summarized in Table 1. A total of 111 patients were included in the study, with a mean age of 70.3 ± 10.5 years, a mean BMI of 28.7 ± 6.1 kg/m², and a male predominance (65%). Age, BMI, height, weight, injected radioactivity and the type or stressor used during [^82^Rb]RbCl-PET/CT were not gender dependent (*p* > 0.05). The most frequent comorbidities were arterial hypertension (80.2%) and dyslipidemia (70.3%), while 31.5% of patients had previously undergone PCI, and 20.7% had a history of myocardial infarction. Nearly 60% of the included patients were on angiotensin-converting enzyme inhibitors (ACE-I), sartans, or sacubitril-valsartan, while 46.8% had a daily intake of beta blockers.

**Table 1:** Patient characteristics, clinical comorbidity, history and medication intake (mean ± SD, BMI=Body mass Index, MBq=Megabecquerel, NYHA = New York Heart association classification of heart failure, COPD=chronic obstructive pulmonary disease, PPH=possible pulmonary hypertension, PCI=percutaneous coronary intervention)

*Patient characteristics*	
Gender (n,%)	Male: 72(64.9%), female: 39(35.1%)
Age (years)	70.3 ± 10.5
BMI (kg/m²)	28.7 ± 6.1
Height (cm)	170.8 ± 9.8
Weight (kg)	83.9 ± 19.3
[^82^Rb]RbCl (MBq)	631 ± 142.7
Pharmacological stressor (n,%)	Adenosine: 66(59.5%), regadenoson: 45(40.5%)
*Clinical categories and history*	
Arterial hypertension (n,%)	89/111 (80.2%)
Diabetes (n,%)	43/111 (38.7%)
Dyslipidemia (n,%)	78/111 (70.3%)
Atrial fibrillation (n,%)	6/111 (5.4%)
NYHA total (n, I/II/III/IV)	20/111 (1/16/2/1)
COPD total (n, I/II/III/IV)	10/111 (3/4/1/2)
PPH (n,%)	18/111 (16.2%)
Lung embolism (n,%)	4/111 (3.6%)
Myocardial infarction (n,%)	23/111 (20.7%)
Aortocoronary bypass (n,%)	6/111 (5.4%)
PCI (n,%)	35/111 (31.5%)
*Medication intake*	
Beta blockers (*n*,%)	52/111 (46.8%)
Diuretics (n,%)	32/111 (28.8%)
ACE-I, sartane, sacubitril-valsartan (n,%)	66/111 (59.5%)
Spironolactone (n,%)	7/111 (6.3%)

### Imaging parameters

CPTT was 9.2 ± 3.2 s at rest, decreasing to 7.6 ± 3.1 s under stress, while NCPTT was 10.6 ± 3.9 beats at rest and 10.9 ± 4.3 beats under stress based on the 3-second frameset. To allow for a more comprehensive methodological comparison with our previous study, data were evaluated using both 10-second and 3-second framesets (Table 2) [12].

**Table 2:** CPTT and NCPTT values derived from dynamic PET imaging, as well as cardiac parameters during 10-second and 3-second timeframes (mean ± SD, HR = heartrate, systolic BP = systolic blood pressure)

*PET derived parameters*		
*10-second frameset*	*rest*	*stress*
CPTT (seconds)	9.7 ± 3.9	8.2 ± 4.3
NCPTT (beats)	11.4 ± 5.3	11.7 ± 6.1
*3-second frameset*	*rest*	*stress*
CPTT (seconds)	9.2 ± 3.2	7.6 ± 3.1
NCPTT (beats)	10.6 ± 3.9	10.9 ± 4.3
*Cardiac parameters*	*rest*	*stress*
HR (b.p.m.)	70.1 ± 13.2	87.9 ± 13.4
systolic BP (mmHg)	132.7 ± 22.3	125.4 ± 26.3
EF_PET_ (%)	58.7 ± 11.1	

The ratio of truncus pulmonalis to aorta ascendens (ratio tr.pulm:aorta) was calculated, resulting in a mean value of 0.8 ± 0.1, with 5 patients demonstrating a ratio ≥ 1.

TTE-derived parameters and dataset completeness are summarized in Table 3. Due to missing data, the analysis focused on EF_Echo_, LA_volume_, TAPSE, E/A_ratio_, and E/e’_ratio_. The mean EF_Echo_, reflecting global LV systolic function, was 57.2 ± 9.6%, while EF_PET_ measured during [⁸²Rb]RbCl-PET/CT averaged 58.7 ± 11.1%. The E/A_ratio_, indicative of LV filling pressure and diastolic function, had a mean of 1 ± 0.7 (n=86). TAPSE, a marker of RV systolic function, averaged 21.7 ± 5 mm (n=93). The E/e’_ratio_, estimating LV filling pressure from mitral inflow (E) and annular tissue velocity (e’), was 11.2 ± 5.1 (n=74). Finally, LA_volume_, reflecting LA size and chronic diastolic dysfunction, averaged 35.1 ± 12.7 mL (n=85).

**Table 3:** Parameters derived from TTE (s’TKA = systolic s’ velocity of tricuspid annulus, RA_area_= right atrial area, LA_diameter_= left atrial diameter, LV ESV = left ventricular end-systolic volume, LV EDV = left ventricular end-diastolic volume, pressure gradient RV-RA = pressure gradient between the right ventricle and the right atrium)

TTE derived parameters	mean ± SD	Missing *n*(%)
EF_Echo_ (%)	57.2 ± 9.6	0
s’TKA (cm/s)	11.5 ± 2.8	70(63%)
TAPSE (mm)	21.7 ± 5	18(16%)
RA_area_ (cm^2^)	16.6 ± 4.2	69(62%)
E/A_ratio_	1 ± 0.7	25(23%)
E/e’_ratio_	11.2 ± 5.1	37(33%)
LA_volume_ (mL)	35.1 ± 12.7	26(23%)
LA_diameter_ (mm)	39.3 ± 7.7	66(60%)
LV ESV (mL)	22 ± 15.1	51(46%)
LV EDV (mL)	49.6 ± 20.2	45(41%)
Pressure gradient RV-RA (mmHg)	27.6 ± 9.9	62(56%)

### Robustness of CPTT and EF_PET_

To assess the reliability and consistency of repeated measurements, ICCs were calculated for EF_PET_ and CPTT during both rest and stress (CPTT_rest+stress_). The ICC for EF_PET_ was 0.806 (95% CI: 0.73–0.86), while CPTT_rest+stress_ showed higher consistency with an ICC of 0.904 (95% CI: 0.83–0.95), indicating strong measurement robustness, particularly for CPTT.

**Fig. 3:**
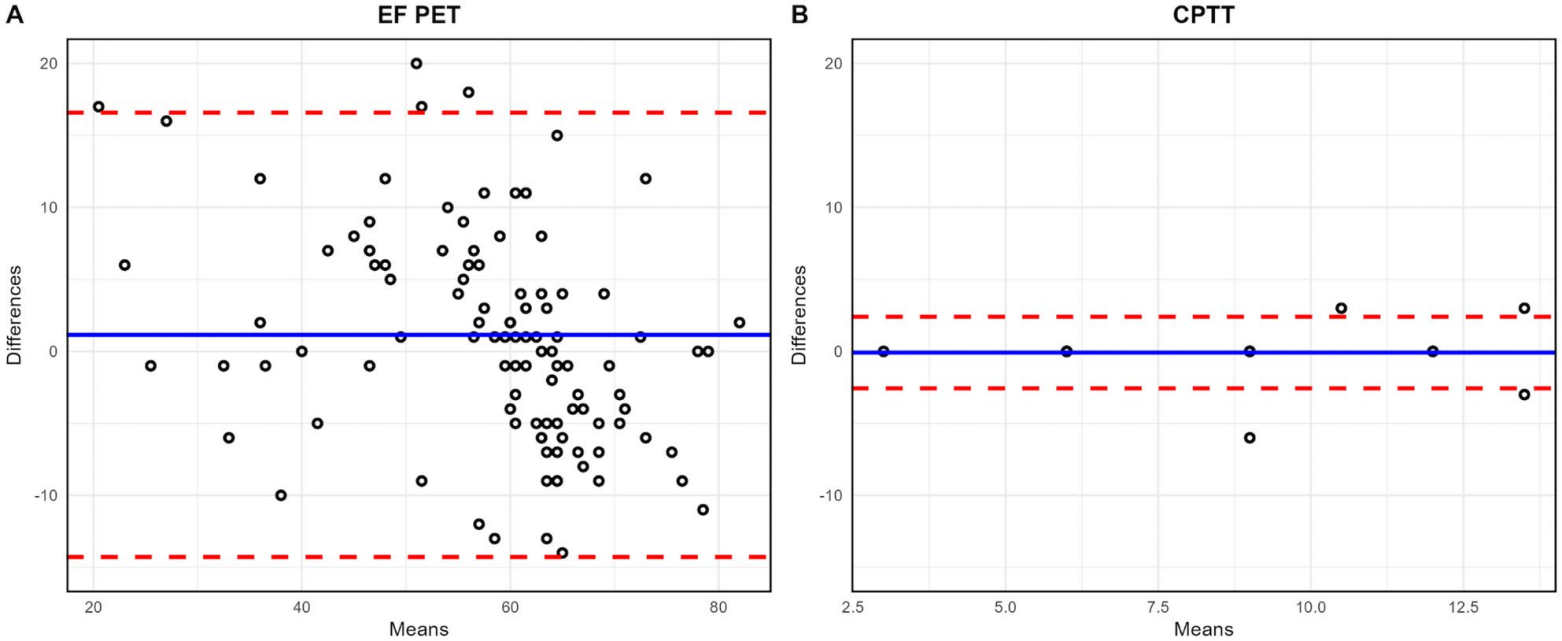
Bland-Altman analysis for EF_PET_ (A) and CPTT_rest+stress_ (B), to assess the agreement between repeated measurements. The mean difference (bias) is represented by the solid blue line, while the 95% limits of agreement are indicated by the dashed red lines. Panel B shows fewer distinct data points due to overlapping values, which explains the denser appearance of the plot

Regarding Bland-Altman analysis, EF_PET_ showed a mean difference of 1.2% with wide limits of agreement (−14.29%;16.58%) and slightly greater variability at higher EF. In contrast, CPTT_rest+stress_ had a mean difference of −0.1 and narrow limits (−2.6;2.4), indicating higher reproducibility (Fig. 3).

### NCPTT and echocardiographic parameters

The linear model explained 26% of the variance in NCPTT_rest_ (R²=0.260, adjusted R²=0.194). Body weight was significantly associated with higher NCPTT_rest_ (β = 0.05, *p* < 0.05), while EF_Echo_ was negatively associated with NCPTT_rest_ (β=−0.08, *p* < 0.05). No significant associations were found for sex, age, height, LA_volume_, TAPSE, E/A_ratio_, E/e’_ratio_ (all *p* > 0.05) (Table 4).

**Table 4:** Linear model with NCPTT_rest_ as a dependent variable, coefficient estimates, standard errors (95%CI = 95% confidence Interval), and p-values for predictors including sex, age, height, weight, EF_Echo_, LA_volume_, TAPSE, E/A_ratio_ and E/e’_ratio_

Predictor	Coefficient estimate	95%CI (lower, upper)	*p*-value
Sex			
Male	-	-	
Female	−0.64	−2.8, 1.5	0.6
Age	−0.01	−0.09, 0.07	0.9
Height	−0.01	−0.12, 0.10	0.9
Weight	0.05	0.01, 0.09	0.024
EF_Echo_	−0.08	−0.17, 0.00	0.045
LA_volume_	0.06	−0.01, 0.13	0.11
TAPSE	−0.09	−0.23, 0.04	0.2
E/A_ratio_	−0.01	−0.21, 0.20	> 0.9
E/e’_ratio_	1.2	−0.16, 2.5	0.084
R^2^	0.260		
Adjusted R^2^	0.194		
AIC	602		
BIC	632		

### NCPTT and types of cardiac dysfunction

Regarding the association between types of cardiac dysfunction and NCPTT_rest_, both systolic RV and LV dysfunction were significantly associated with NCPTT_rest_ (OR = 1.21, 95%CI:1.04–1.42, *p* < 0.05, and OR = 1.18, 95%CI:1.04–1.34, *p* < 0.01, respectively). Diastolic LV dysfunction showed no significant association with NCPTT_rest_ (OR = 1.00, 95%CI:0.88–1.14, *p* > 0.05). Increased PA pressure was associated with significantly higher NCPTT_rest_ odds (OR = 1.21, 95%CI:1.02–1.44, *p* < 0.05)(Table 5).

**Table 5:** Univariate logistic models and calculated the odds ratios (OR) with corresponding confidence intervals (CI) and p-values to assess associations between types of cardiac dysfunction and NCPTT_rest_

Types of cardiac dysfunction	OR	Lower CI	Upper CI	*p*-value
Diastolic LV Dysfunction	1.00	0.88	1.14	0.969
Systolic RV Dysfunction	1.21	1.04	1.42	0.011
Systolic LV Dysfunction	1.18	1.04	1.34	0.009
Increased PA Pressure	1.21	1.02	1.44	0.028

### NCPTT in relation to patient history and clinical categories

Looking into the relationship of NCPTT_rest_ with patients’ history and clinical categories, a significant association between NCPTT_rest_ and PPH (*p* < 0.05) could be shown. All other variables showed no significant associations (Table 6).

**Table 6:** Number of distinct clinical categories and the presence of medical history, along with the corresponding NCPTTrest values (in italics, mean ± SD). Results from Wilcoxon rank-sum tests are presented, along with the corresponding p-values indicating statistical significance

Clinical categories	no *n* (NCPTTrest: mean ± SD)	yes *n* (NCPTTrest: mean ± SD)	*p*-value
Diabetes	67 *(10.5 ± 3.2)*	44 *(10.7 ± 4.7)*	0.861
NYHA > 0	91 *(10.7 ± 3.7)*	20 *(10.3 ± 4.7)*	0.683
Arterial hypertension	22 *(10.5 ± 4)*	89 *(10.6 ± 3.8)*	0.877
Dyslipidemia	33 *(10 ± 2.8)*	78 *(10.9 ± 4.2)*	0.268
PPH	93 *(10.2 ± 3.7)*	18 *(12.6 ± 4.4)*	0.016
COPD (GOLD) > 0	101 *(10.7 ± 3.9)*	10 *(9.2 ± 3.4)*	0.219
*Medical history*			
Lung embolism	107 *(10.7 ± 3.9)*	4 *(9.1 ± 3.4)*	0.431
Myocardial infarction	88 *(10.4 ± 3.7)*	23 *(11.4 ± 4.3)*	0.295
Aortocoronary bypass	105 *(10.6 ± 3.9)*	6 *(10.4 ± 2.8)*	0.879
PCI	76 *(10.3 ± 3.8)*	35 *(11.3 ± 4)*	0.215

### The H_2_FPEF score & Cut-Off value for NCPTT_rest_

**Fig. 4:**
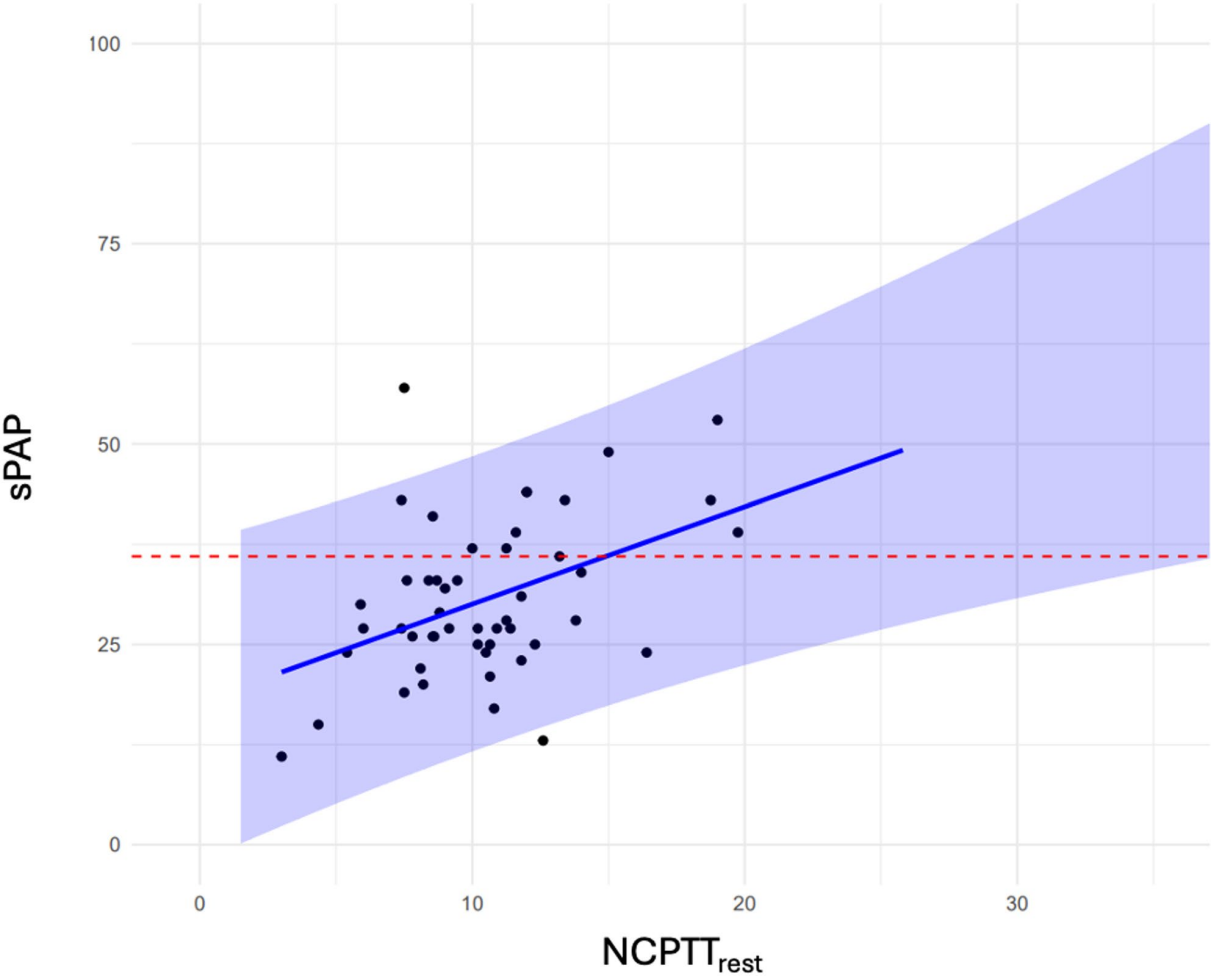
Linear regression: Association between sPAP and NCPTT_rest_. The dotted red line indicates a sPAP of 36mmHg

HFpEF was ruled out (H_2_FPEF score < 2) in 21.6% (*n* = 24); 75.7% had scores between 2 and 5, requiring further testing, and 2.7% (*n* = 3) had scores ≥ 6, supporting the diagnosis. Linear regression showed a significant association between sPAP and NCPTT_rest_ (*p* < 0.01); an sPAP of 36 mmHg corresponded to an NCPTT_rest_ of ~ 15 beats (Fig. 4).

## Discussion

This study demonstrates that NCPTT_rest_, derived from routine [⁸²Rb]RbCl-PET/CT using a refined 3-second frame protocol, is a robust and reproducible marker that correlates with key indicators of cardiac function, most notably RV and LV systolic dysfunction and elevated PA pressure, as well as PPH. These associations position NCPTT_rest_ as a promising non-invasive tool for the identification of patients with PPH. Also, NCPTT_rest_ could help in stratifying patients using the H_2_FPEF score. This is a new finding compared to our previous study [12]. 

Our results showed that higher NCPTT_rest_ was significantly associated with lower EF_Echo_ (β=−0.08, *p* < 0.05). This is in line with data from our previous research (β=−0.77, *p* < 0.01) [12]. The association between CPTT and NCPTT with LVEF has been extensively investigated in various cardiac imaging modalities, demonstrating consistent findings across multiple studies [9, 10, 16–19]. A prospective study including 57 patients with heart failure with reduced ejection fraction (HFrEF) evaluating the relationship between normalized pulmonary transit time (nPTT, ‘equivalent’ to NCPTT in our study) and cardiac function, as well as pulmonary hemodynamics could demonstrate a significant negative correlation between nPTT and ventricular ejection fraction, with a stronger correlation to right ventricular ejection fraction (RVEF) compared to LVEF (*r*=−0.77, *r*=−0.67, *p* < 0.001) [17]. A retrospective study, including 352 patients referred for a routine stress perfusion MRI, demonstrated a significant negative association between PTT and LVEF with significantly higher PTT values in patients with reduced LVEF (< 40%) compared to preserved LVEF (≥ 40%) (9.3 vs. 7.1 s; *p* < 0.001) [10]. 

Furthermore, when categorizing types of cardiac dysfunction based on echocardiographic data, we showed that elevated NCPTT_rest_ was significantly associated with both LV and RV systolic dysfunction, as well as increased PA pressure, whereas diastolic LV dysfunction does not appear to influence NCPTT_rest_ outcomes. These findings support our previously discussed observation of a negative correlation between NCPTT_rest_ and LVEF, a key marker of LV systolic function. Our results are also consistent with a prior prospective study involving 112 patients with chronic heart failure, which reported that impaired biventricular systolic function correlated with the pulmonary blood volume index, a parameter shown to be proportional to PTT [16]. 

Our findings demonstrate a significant association between NCPTT_rest_ and the presence of PPH (*p* < 0.05), as well as parameters indicative of PH, such as sPAP (*p* < 0.01), or as demonstrated in the previous section by types of heart dysfunction, increased PA pressure (*p* < 0.05). This aligns with prior research highlighting the diagnostic potential of PTT in PH detection. PH is potentially life-threatening and especially post-capillary group 2 with left ventricular heart failure is of clinical relevance in patients with chronic coronary artery disease. It is of interest to limit the need for RHC performed for diagnostic purposes in patients most likely belonging to PH groups 2, 3 and 5, as complications with this invasive procedure might occur [20, 21]. Specifically, previous studies have shown a significant positive correlation between PTT and established hemodynamic markers of PH [9, 16, 22], including pulmonary artery wedge pressure (PAWP) as well as mPAP (*r* = 0.74, *r* = 0.68), underlining PTT as a tool to detect PH [9]. 

PH is commonly observed in patients with HFpEF as well as HFrEF [1, 23–26], with heart failure itself being one of the leading causes of secondary PH [27, 28]. An observational study involving 244 patients with HFpEF (LVEF ≥ 50%) revealed that 83% of the cohort exhibited PH, defined as pulmonary artery systolic pressure (PASP, equivalent to sPAP in our study) > 35mmHg, as measured by echocardiography. PH was significantly associated with increased mortality, with each 10mmHg rise in PASP corresponding to a hazard ratio (HR) of 1.3 (*p* < 0.001) [23]. Similarly, in a cohort of 196 patients with HFrEF (LVEF 18–36%), PH at time of diagnosis was associated with an adjusted risk ratio of 2.3 (95%CI:1.42,3.73) for acute heart failure or death (*p* < 0.001) [29]. A study focusing on RHC, as the gold standard in detecting PH, has further elucidated the impact of PH in heart failure, revealing that PH is prevalent in HFrEF and that each 5 mmHg increase in mPAP is associated with a 10% increase in mortality risk (*p* < 0.001) or a HR of 2.2 for death (*p* < 0.001) [24, 29], particularly in precapillary PH [25]. 

Given the well-established association between PH and heart failure, recent research has investigated the potential of PTT not only in PH detection but risk stratification across a spectrum of cardiac conditions, including heart failure [18, 22, 30]. The likelihood of HFpEF in our cohort was assessed using the H_2_FPEF score [13]. A definitive diagnosis was established in approximately 3% of patients, while ~ 76% fell into the intermediate probability range, warranting further diagnostic evaluation. Since sPAP and E/e’ contribute to the score and given the incomplete data in our cohort, the actual diagnostic likelihood may be underestimated. Notably, the significant association observed between NCPTT_rest_ and sPAP suggests that NCPTT_rest_ could support the interpretation of the H₂FPEF score, with an sPAP of 36 mmHg corresponding to an NCPTT_rest_ of approximately 15 beats.

Supporting the clinical relevance of PTT, a prospective study including 506 patients demonstrated that prolonged PTT is associated not only with a significantly increased risk of heart failure but also significantly higher long-term risk of heart failure hospitalization. Specifically, nPTT ≥ 8.2 was associated with an adjusted HR for heart failure of 3.69 (95%CI:1.09,1.27), and each 1-unit increase of nPTT corresponded to an HR of 1.26 (95%CI:1.18,1.33) [18]. Another study focusing on cardiac MRI derived PTT aligns with the statement proving PTT to be a predictor of outcomes in heart failure, including heart failure hospitalization (HR1.15, 95%CI:1.02,1.29, *p* = 0.02) [30]. 

At last, there is a clear benefit in reducing the temporal resolution from previously 10 s to 3 s. The frameset duration refers to the length of each time interval during data acquisition. It directly influences temporal resolution, as shorter frames can more accurately capture rapid physiological changes, which lead to an enhanced data quality and precision. In comparison, longer frame sets improve statistical noise characteristics. Noteworthy, other imaging modalities have a temporal resolution of approximately 1 s for MRI and below 100 msec for modern CT scanners [10, 31]. 

Limitations.

This study’s retrospective nature led to incomplete echocardiographic data, which could be improved by a prospective design. Furthermore, as NCPTT has not yet been validated against the gold standard of right heart catheterization, the present findings should be regarded as preliminary and warrant prospective confirmation. In addition, only 18 of 111 patients had PPH, underscoring the need for larger cohorts. Also, while the temporal resolution was optimized by shortening the timeframes, even shorter intervals could further enhance precision. Finally, manual placement of VOIs in the ventricles carries an inherent risk of user-dependent variability. To minimize this, repeated measurements were performed, and excellent consistency was confirmed using ICC. Nevertheless, the implementation of standardized, automated VOI placement, potentially powered by machine learning algorithms, could further reduce variability and enhance reproducibility. This would be particularly valuable given the growing body of evidence supporting the clinical utility of NCPTT as a non-invasive marker in both cardiovascular and pulmonary diagnostics.

## Conclusion

In conclusion, our findings demonstrate the diagnostic and prognostic value of NCPTT_rest_ as a useful additional marker for assessing cardiac function and could help in the evaluation of patients with suspected cardiac systolic dysfunction as well as the detection of PH. Moreover, its integration into the H₂FPEF score may enhance patient stratification, underscoring its potential utility in routine cardiovascular assessment and management.

## Supplementary Information

Supplementary Material 1

Supplementary Material 2

Supplementary Material 3

Supplementary Material 4

## Data Availability

The datasets used and analyzed during the current study are available from the corresponding author on reasonable request.

## Abbreviations

ACE-I: Angiotensin-converting enzyme inhibitor
COPD: Chronic obstructive pulmonary disease
CPTT: Cardiopulmonary transit time
DHARMa: Residual Diagnostics for Hierarchical Models
E/A_ratio_: E-Wave to A-Wave ratio
E/e’_ratio_: Mitral inflow velocity (E) and mitral annular tissue velocity (e’) ratio
EF: Ejection fraction
EF_Echo_: Left ventricular ejection fraction derived from TTE
EF_PET_: Left ventricular ejection fraction derived from PET
HFpEF: Heart failure with preserved ejection fraction
HFrEF: Heart failure with reduced ejection fraction
ICC: Intraclass correlation coefficient
LA_volume_: Left atrial volume
LV: Left ventricle
LVEF: Ejection fraction of the left ventricle
mPAP: Mean pulmonary arterial pressure
MRI: Magnetic resonance imaging
NCPTT: Normalized cardiopulmonary transit time
NCPTT_rest_: Normalized cardiopulmonary transit time during rest
NCPTT_stress_: Normalized cardiopulmonary transit time during stress
NYHA: New York Heart Association classification of heart failure
PA: Pulmonary arterial
PCI: Percutaneous coronary intervention
PET/CT: Positron emission tomography/Computed tomography
PH: Pulmonary hypertension
PPH: Possible pulmonary hypertension
RAP: Right atrial pressure
[^82^Rb]RbCl: Rubidium-chloride
RHC: Right heart catheterization
RV: Right ventricle
RVEF: Ejection fraction of the right ventricle
sPAP: Systolic pulmonary artery pressure
SUV: Standardized uptake value
TAPSE: Tricuspid annular plane systolic excursion
TTE: Transthoracic echocardiography
VOI: Volume of interest
