# Supplementary material for: Cardiopulmonary transit time measured on dynamic rubidium cardiac PET/CT is a predictor for pulmonary hypertension
Source: EJNMMI Res. 2026 Jun 22;16:119. doi: 10.1186/s13550-026-01387-y (PMC13433693; doi:10.1186/s13550-026-01387-y)
Supplement: Supplementary file 1 [file 13550_2026_1387_MOESM4_ESM.docx]

Supplementary Materials

## Materials and Methods: Study design and population

## Methodology Comparison: 10 second vs 3 second frameset

## Compared to the previous study (supp. 1), framesets during [⁸²Rb]RbCl-PET/CT were reduced from 10 to 3 seconds to enhance temporal resolution and data quality. Density histograms of both settings are shown (Supplementary Figure 1), illustrating smoother distributions with 3-second frames and eliminating impossible NCPTT values of zero. An example is shown (Supplementary Figure 2) of a 57 year old patient, illustrating the effect of temporal resolution on SUVmean time-activity curves. The 3-second frameset produces a sharper peak compared to the 10-second frameset, allowing more precise CPTT measurement. NCPTTrest was 15 beats (CPTTrest = 12 s) with the 3-second frameset, versus 25 beats (CPTTrest = 20 s) with the 10-second frameset.

## *Supplementary Figure 1: Comparison of NCPTT_rest_ values at 3 seconds and 10 seconds. The histograms are overlaid with blue density curves to illustrate the smoothness and variation in measurement precision between the measurement intervals.*

## *Supplementary Figure 2: SUV_mean_ time-activity curves of the right ventricle (VOI_RV) and the left ventricle (VOI_LV) during rest, evaluated over both 10-second and 3-second framesets.*

H_2_FPEF-Score

*Supplementary Figure 3: H_2_FPEF-Score*

Patient History and Clinical Categories

The comorbidities recorded included arterial hypertension, diabetes, dyslipidemia, atrial fibrillation during [^82^Rb]RbCl-PET/CT, heart failure (classified by the NYHA functional classification), chronic obstructive pulmonary disease (COPD), pulmonary hypertension and the history of lung embolism, myocardial infarction, aortocoronary bypass surgery and percutaneous coronary intervention (PCI). Current medications, such as beta blockers, diuretics, angiotensin-converting enzyme inhibitors (ACE-I)/ sartane/ sacubitril-valsartan, spironolactone, were also documented. All clinical data were extracted from the institution’s internal patient information system. Arterial hypertension was recognized not only based on prior diagnoses but also in accordance with established guidelines.(supp. 2)

# Supplementary References

supp. 1	Seige LC, Zhang B, Heimer J, Spielhofer N, Popescu C, Murray K, et al. Is cardiopulmonary transit time (CPTT) measured by using dynamic rubidium cardiac PET/CT a predictor for cardiac function? Int J Cardiovasc Imaging. 2025 Feb 14;41(3):569–77.

supp. 2	Bakris G, Ali W, Parati G. ACC/AHA Versus ESC/ESH on Hypertension Guidelines. J Am Coll Cardiol. 2019 Jun;73(23):3018–26.
